# 

**Published:** 1894-06-02

**Authors:** 


					I- Br,,.- >riii/\nrlLirtr ^ ^ -<SF W sIZ WITH EXTRA NURSING SUPPLEMENT.
'? e?.TwPPE.N9i^> Ur v*t ~ - - -
ff PRICE TWOPENCE. ssj^ WW W. wl
?.401, Vol. XVI.?June 2nd, 1894. HP IhI ll
?t the General Post Office as a Newspaper for )??> <-?-1 r^!r i V J
^mission in the United Kingdom and Abroad.!
Per Annum, 8s. 6d., post free.
Monthly Farts, 6d.; post free, 8d.
osmisaion in the United Kingdom and Abroad*] ~ Ditto per Annum, 8s.. post free.
'RWICH HOSPJ.TAL
No. 401 Vol. xti, WITH EXTRA NURSING SUPPLEMENT. Saturday,
June 2nd, 1894.

				

